# Gene delivery and gene expression in vertebrate using baculovirus *Bombyx mori* nucleopolyhedrovirus vector

**DOI:** 10.18632/oncotarget.22522

**Published:** 2017-11-20

**Authors:** Xingjian Liu, Yinü Li, Xiaoyuan Hu, Yongzhu Yi, Zhifang Zhang

**Affiliations:** ^1^ Biotechnology Research Institute, Chinese Academy of Agricultural Sciences, Beijing, China; ^2^ The Sericultural Research Institute, Chinese Academy of Agricultural Sciences, Zhenjiang, China

**Keywords:** *Bombyx mori* nucleopolyhedrovirus, complement resistance, gene delivery

## Abstract

The baculovirus *Autographa californica* multicapsid nucleopolyhedrovirus (*Ac*MNPV) has been investigated as a possible tool for gene therapy, but its inhibition by complement proteins in human serum limits its applicability. Here, we used the baculovirus *Bombyx mori* nucleopolyhedrovirus (*Bm*NPV) to construct a gene delivery vector in which a reporter gene is driven by a cytomegalovirus IE promoter. Enhanced green fluorescent protein (EGFP) and luciferase reporter genes were used to test the efficiency of gene delivery. *In vitro* complement inactivation data showed that the recombinant *Bm*NPV vector was more stable in human serum than the recombinant *Ac*MNPV vector. The recombinant *Bm*NPV vector successfully delivered the reporter genes into different tissues and organs in mice and chicks. These results demonstrate that the *Bm*NPV vector is more stability against complement inactivation in human serum than the *Ac*MNPV vector, and indicate that it may be useful as an effective gene delivery vector for gene therapy in vertebrates.

## INTRODUCTION

The idea of gene therapy was originally conceived in the 1970s [1, 2]. The feasibility was later confirmed by experiments in mice [3], and the first clinical application in humans was performed in the 1990s [4]. The method continues to show promise for treating inherited diseases and cancer [5–7].

The gene delivery system used to deliver nucleic acids into target cells is a key component in determining the clinical success of a gene therapy treatment. The most efficient delivery systems are based on viruses that infect animal cells, such as lentiviruses, retroviruses, adenoviruses and adeno-associated viruses [8]. Baculoviruses have been also explored as a possible gene delivery vector system for vertebrate cells [9–12]. These viruses may be ideal for gene therapy because their viral promoters are almost silent in mammalian cells, and the budded viral form is harmless to the environment [13–16]. *Autographa californica* multicapsid nucleopolyhedrovirus (*Ac*MNPV), a widely studied baculovirus, is as a promising gene therapy vector [17]. Previous *in vivo* studies have demonstrated that *Ac*MNPV directly injected into target tissues, including brain and testis, can drive the foreign gene expression in rats and mice [18–21]. A single intravitreal injection of recombinant baculovirus could induce target gene expression in eye [22, 23]. In addition, the *Ac*MNPV vector has been investigated as a possible tool for cancer gene therapy [24–26]. However, *Ac*MNPV is inhibited by the complement proteins in human serum, which restricts its applications for gene delivery *in vivo* [27].

*Bombyx mori* nucleopolyhedrovirus (*Bm*NPV) is another baculovirus with a limited host range [28, 29]. This virus also drives the expression of target genes in mammalian cells [30]. In this study, we used *Bm*NPV-derived recombinant baculoviruses to deliver reporter genes into mice and chicks. The cytomegalovirus (CMV) IE promoter [31] was chosen to drive reporter gene expression *in vivo*. Our *in vitro* and *in vivo* results demonstrate that the *Bm*NPV vector exhibits a greater stability against complement inactivation in human serum than the *Ac*MNPV vector.

## RESULTS

### Construction of reporter genes delivery vectors

**Figure 1:**
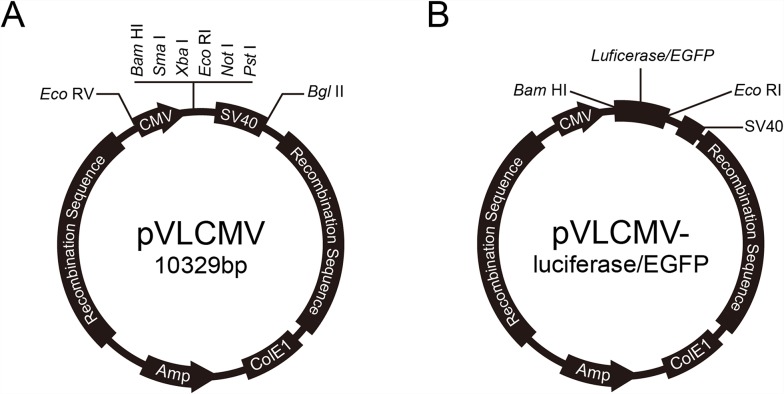
Reporter genes transfer vector

We constructed Baculovirus transfer vectors containing CMV IE promoter based on the pVL1393 vector. The polyhedron promoter of the pVL1393 transfer vector was replaced with the CMV IE promoter. SV40 polyadenylation signal was inserted downstream the multiple cloning site. The CMV promoter transfer vector was named pVLCMV (Figure 1A). Luciferase and enhanced green fluorescent protein (EGFP) genes were separately inserted into the vector (Figure 1B). The recombinant baculoviruses reBm-luc and reBm-EGFP, used to deliver the luciferase and EGFP genes, respectively, were prepared by using cotransfection of reBmBac and pVLCMV-luc or pVLCMV-EGFP in Bm cells. The recombinant baculovirus vector reAc-luc was used for comparison assays of complement inactivation. The vector was prepared by cotransfection of BacPAK6 and pVLCMV-luc.

Fifth instar silkworm larvae and Sf9 cells were infected with the recombinant baculoviruses for amplification. The recombinant baculoviruses vectors were amplified in larval hemolymph and cell-culture supernatant, purified, and concentrated by ultracentrifugation. Purified viruses were quantified by real-time PCR (Q-PCR) to determine the amount of viral genome per milliliter (vg/mL). The quantified results for the reporter genes were consistent with those of the viral genome, which made it possible to determine the purity of the recombinant viruses and number of copies of the viral genome.

### Resistance to complement inactivation

**Figure 2:**
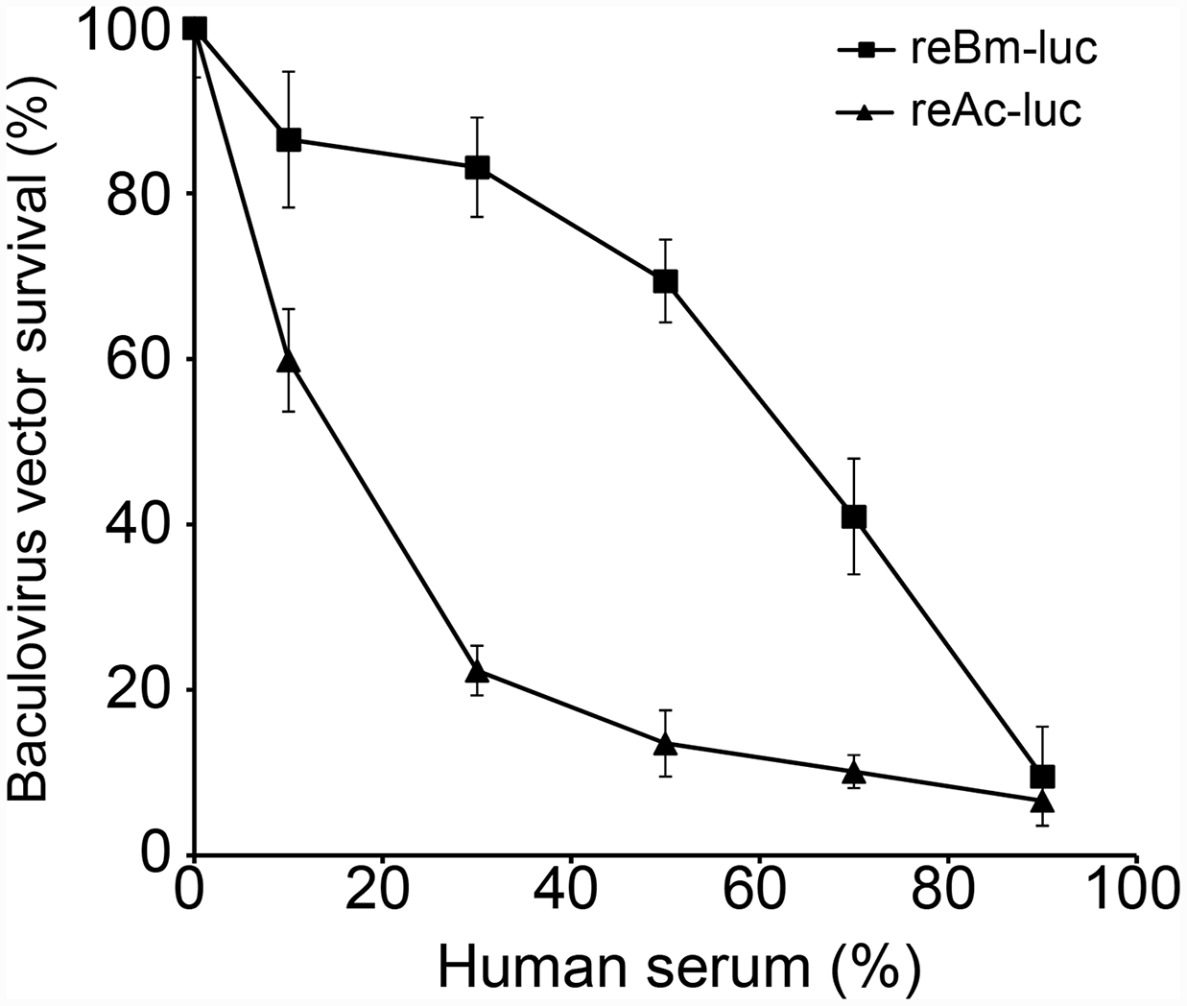
Baculovirus survival in human serum Recombinant *Bm*NPV showed stronger resistance to complement inactivation in human serum than recombinant *Ac*MNPV.

The reBm-luc recombinant baculovirus was used to measure resistance to complement inactivation in human serum. Compared with luciferase gene delivery *Ac*MNPV (reAc-luc), reBm-luc exhibited increased stability in human serum (Figure 2). When the serum concentration reached 50%, the survival rate of reBm-luc was approximately 70%, whereas reAc-luc exhibited 16% survival. These results somewhat differ from a previous report by Hofmann *et al* [26]; this difference might have been caused by technical differences and different cells used. The results for *Ac*MNPV were consistent.

### EGFP gene expression *in vitro* and *in vivo*

VERO, PK-15, HEK293T, and Hela cells can be transduced by recombinant baculovirus. Here, we used these cell lines to verify the gene delivery *in vitro*. Fluorescence imaging of reBm-EGFP transduced cells shows the EGFP expression (Supplementary Figure 1).

**Figure 3:**
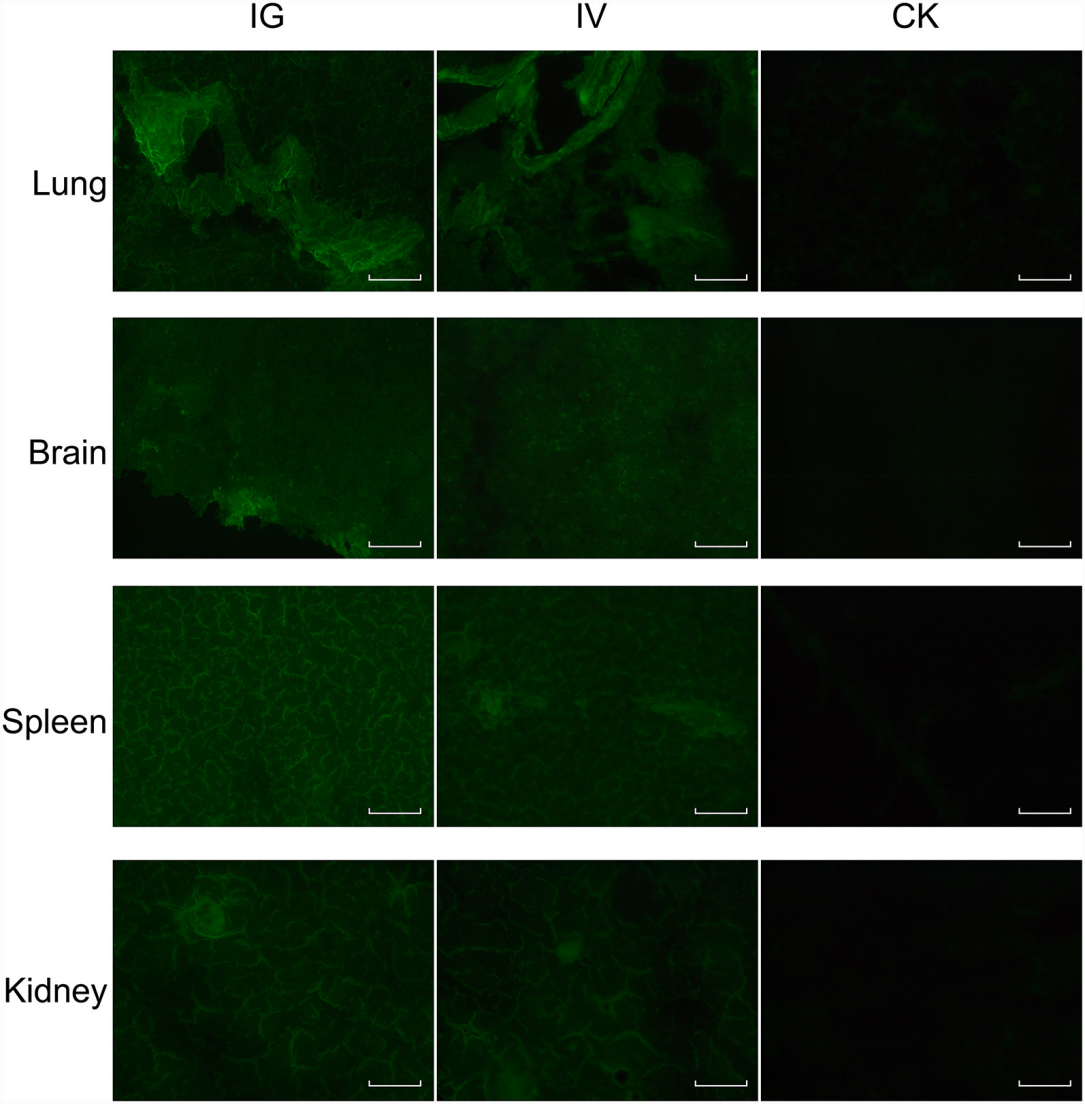
EGFP expression in mice Abbreviations: IG, intragastric administration group; IV, intravenous injection group; CK, control group. Scale bar, 100 μm.

**Figure 4:**
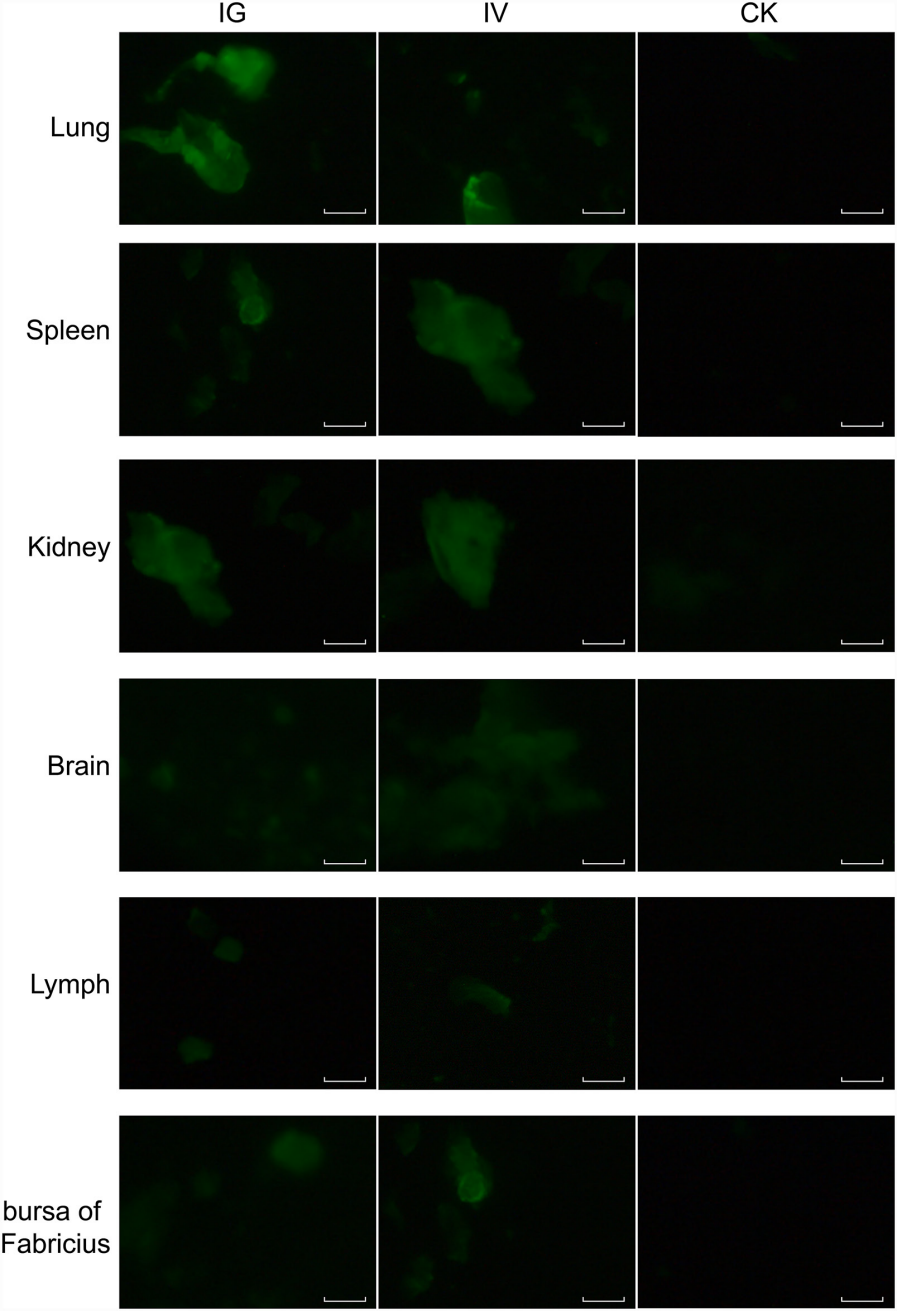
EGFP expression in chicks Abbreviations: IG, intragastric administration group; IV, intravenous injection group; CK, control group. Scale bar, 100 μm.

**Figure 5:**
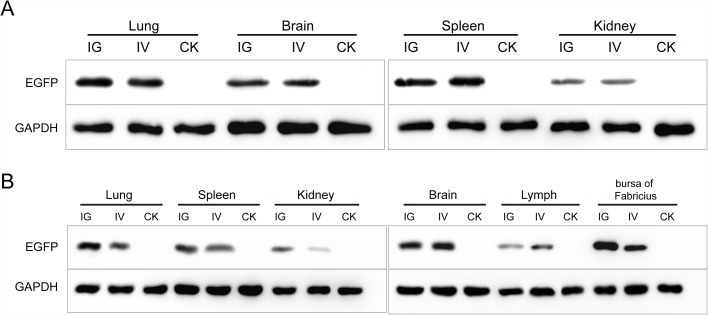
The Western blotting analysis of EGFP in mice **(A)** and chicks **(B)**. Abbreviations: IG, intragastric administration group; IV, intravenous injection group; CK, control group.

The recombinant baculovirus reBm-EGFP was used to transfect mice and chicks. Fluorescence imaging of mouse tissue slices indicated that EGFP was effectively expressed in the lungs, spleens, kidneys, and brains (Figure 3) from the 5^th^ day to the 17^th^ day after intravenous injection (IV) or intragastric administration (IG). Immunoblotting analysis verified the protein expression of EGFP-constructs in these organs (Figure 5A). Similar results were observed in the lungs, spleens, kidneys, bursa of Fabricius, and brains of chicks (Figure 4 and Figure 5B). After the 21st day, no EGFP expression was observed in the above tissues in mice and chicks. However, in the intramuscular injection (IM) groups, EGFP was not detected. In addition, the fluorescence background in liver was too strong to identify any specific EGFP expression. Due to the presence of fluorescence in most tissues, a luciferase reporter gene delivery assay was performed to verify the EGFP results.

### Luciferase gene expression *in vivo*

**Figure 6:**
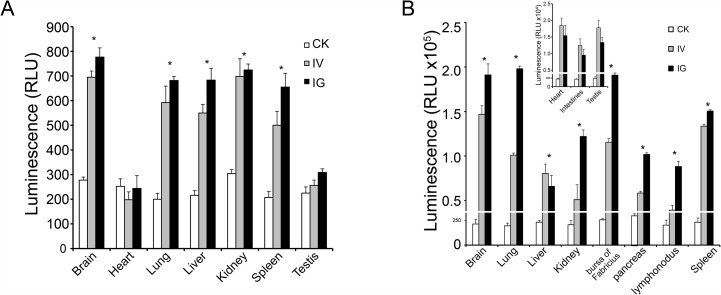
Luciferase activity *in vivo* **(A)** Luciferase activity was detected in mouse brain, lung, liver, kidney and spleen. **(B)** Luciferase activity was detected in most tissues in chicks. The expression efficiency in chicks was higher than in mice.

Mice and chicks tissues were collected and assayed for luciferase activity at 5, 11, 17, 21, and 30 days after reBm-luc intravenous (IV), intramuscular (IM), or intragastric (IG) administration. In the mouse IV and IG groups, luciferase activity was observed in lung, liver, kidney, spleen, and brain at 5 and 11 days (Figure 6A). No significant luciferase activity was detected in these tissues after 17 days. In chicks of the IV and IG groups, luciferase activity was detected in lung, liver, kidney, bursa of Fabricius, pancreas, lymphonodus, and brain at 5 and 11 days (Figure 6B). After 17 days, the luciferase activity disappeared. No luciferase activity was detected in mouse muscle tissues. However, a weak activity was detected in chicks of the IG and IV groups. No luciferase expression was detected in mice or chicks IM groups.

## DISCUSSION

Our results indicate that the *Bm*NPV vector is more stable and resistant to inactivation by the human serum complement compared with the *Ac*MNPV vector. The *Ac*MNPV baculovirus vector has been widely used for gene delivery, but it is complement-sensitive [26]. Thus, in the gene therapy field, baculoviruses have been used only to deliver target genes into tissues in which the viruses are not exposed to complement proteins [19, 20, 22, 32], and to prepare recombinant adeno-associated virus (rAAV)-containing target genes [33, 34]. Numerous gene therapy trial *in vivo* studies have used the *Ac*MNPV vector, even in cancer gene therapy [24–26], but its complement sensitivity restricts its further clinical use. Many strategies have been devised to overcome the *Ac*MNPV complement sensitivity. One approach uses fusion proteins that provide protection against complement inactivation by displaying them on the *Ac*MNPV viral envelope [35–37]. In the *Bm*NPV vector, the surface structure of baculovirus BV (budded virus) may be a key factor responsible for the increased resistance to complement inactivation. *Bm*NPV might serve as an alternative strategy that uses baculovirus vector in gene therapy applications.

Our EGFP and luciferase reporter gene delivery data in mice and chicks confirm that recombinant *Bm*NPV exhibits complement resistance and can deliver foreign genes *in vivo*. In mice, luciferase expression was detected in many important organs: lung, liver, kidney, spleen, and brain. EGFP was also detected in these organs, except in liver, which exhibited fluorescence background too high to allow detection of EGFP fluorescence. To overcome this limitation, we also used a luciferase reporter gene. In chicks, the expression of the reporter genes was similar to mice. Additional expression was observed in the bursa of Fabricius. The luminescence and immunoblotting data showed that the luciferase expression was different in different tissues, indicating that the gene delivery effectiveness of *Bm*NPV differs among different tissues. The underlying mechanisms may involve a certain degree of complement inactivation, differences in baculovirus distribution, and its different half-lives in different tissues. Complement inactivation is still a key factor determining the success of baculovirus-based gene delivery [27]. In different tissues, exposure times of baculovirus to complement are different. Since *Bm*NPV vectors, like other gene delivery vectors, are exogenous and cannot replicate *in vivo*, they are cleared-up in tissues [38]. Thus, different organs and tissues differ in the distribution of baculovirus and other foreign genes.

Our luminescence results indicate that the luciferase expression efficiency in chicks is higher than in mice. The luciferase activity was detected in the heart, intestines, and testes of chicks, but it was not detected in those tissues in mice. The duration of recombinant *Bm*NPV-driven reporter gene expression in animals was shorter than that of recombinant *Ac*MNPV-driven expression in cells [30]. In addition, 17 days after virus infection, reporter gene expression disappeared because the baculovirus cannot replicate in animal cells [14], and its genomic DNA is less stable in tissues compared with cells. This feature ensures biosafety and makes the virus harmless to healthy animals and humans.

In IG and IV groups, EGFP and luciferase were detected in both mice and chicks. Our luminescence and Western blotting results showed that the tissue reporter gene expression was higher in the IG groups compared to the IV groups. However, in the IM groups, expression of EGFP and luciferase reporter genes was not detected. According to a previously published study [39], baculovirus-mediated gene delivery might be less efficient when administered via muscle. Other mechanisms may be involved in addition to the complement inactivation effect. By different administration routes, gene delivery vectors may enter the systemic circulation and be distributed to tissues through different pathways and with different absorption efficiencies [40].

The baculovirus system can be used for expression *in vitro* and *ex vivo*, and may also be effective for gene therapy *in vivo*. Early attempts were made to use the *Ac*MNPV vector for transduction in mice. However, our results demonstrate that the *Bm*NPV vector exhibits better characteristics for gene delivery in vertebrates. Additional modifications, such as surface shielding with decay acceleration factor, may help improve the efficiency of the recombinant *Bm*NPV vector. Furthermore, the copy numbers of recombinant *Bm*NPV viral genomes produced in one silkworm could reach 10^12^ vg, which provides higher efficiency for the production of recombinant baculovirus for gene delivery. The virtual inactivity of baculoviral promoters in vertebrate cells and their non-pathogenic nature in vertebrates ensure their safety for use in gene delivery. Our results indicate that the *Bm*NPV vector exhibits a strong potential for gene therapy research and clinical applications.

## MATERIALS AND METHODS

The study was conducted in accordance with animal ethics guidelines. All animal procedures were approved by the Beijing Administration Office of Laboratory Animals. The *Bombyx mori*-derived cell line *Bm*5 was cultured in TC100 insect cell culture medium (Applichem, Darmstadt, Germany) with 10% fetal bovine serum (Gibco, USA) at 27°C. VERO, PK-15, and Hela cells were cultured in Dulbecco's Modified Eagle Medium (DMEM, Gibco, USA) with 10% fetal bovine serum (FBS) at 37°C. HEK293T cells were cultured in Minimum Eagle medium (MEM, Gibco, USA) with 10% FBS at 37°C. *E. coli* Top10 cells were obtained from Invitrogen (Carlsbad, USA), and competent cells were prepared as described [41]. Eight-week-old C57BL/6 mice and 15-day-old specific pathogen-free (SPF) chicks were obtained from Vitalriver (Beijing, China). Human serum was obtained from Sigma-Aldrich (Deisenhofen, Germany). The reBmBac vector was constructed and prepared in our lab [42]. Expression of EGFP was analyzed by western blotting using the rabbit polyclonal anti-EGFP antibody (ab6556, Abcam, UK).

### Preparation of gene delivery viruses

The CMV promoter was PCR-amplified (CMV-F: GGATATCTAGTTATTAATAGTAATCAATTACGG; CMV-R: CGGATCCGGATCTGACGGTTCACTAAACCAGC) from the pEGFP-N3 vector (Invitrogen, Carlsbad, USA) and inserted into the pVL1393 transfer vector (Invitrogen) by *Eco*RV-*Bam*HI digestion. Meanwhile the polyhedron promoter in pVL1393 was replaced. The SV40 polyadenylation signal was then PCR-amplified (SV40-F: TGCGGCCGCACTGCAGTCATAATCAGCCATACCACATTTGT; SV40-R: GAGATCTACATTGATGAGTTTGGACAAACC) from the pEGFP-N3 vector and inserted into the edited pVL1393 vector downstream of the CMV promoter by *Not*I-*Bgl*II digestion. The CMV promoter and SV40 polyadenylation signals were added to the transfer vector, which was named pVLCMV. The luciferase reporter gene was PCR-amplified (Luc-F: CGGATCCATGGAAGACGCCAAAAAC; Luc-R: GGAATTCTTACACGGCGATCTTTCCGC) from the pGL3 vector (Promega, USA) and subcloned into the pVLCMV vector by *Eco*RI-*Bam*HI digestion. This reaction produced the luciferase reporter gene transfer plasmid, which was named pVLCMV-luc. Similarly, the EGFP reporter gene transfer plasmid, pVLCMV-EGFP, was prepared by cloning the EGFP gene (EGFP-F: CGGATCCATGGTGAGCAAGGGCGAGGAG; EGFP-R: GGAATTCTTACTTGTACAGCTCGTCCATG) from pEGFP-N3 vector (Clontech, USA).

The recombinant *Bm*NPVs for reporter gene delivery, reBm-luc and reBm-EGFP, were prepared by co-transfection in *Bm*5 cells with reBmBac and pVLCMV-luc or pVLCMV-EGFP as described [42, 43], and purified by plaque screening [44]. Fifth instar silkworm larvae were used to amplify the viruses; they were injected with recombinant *Bm*NPVs (approximately 10^5^ plaque forming units, p.f.u.). The recombinant *Ac*MNPV reAc-luc, which was used to deliver the luciferase gene, was constructed in Sf9 cells by cotransfection of BacPAK6 (Clontech) and pVLCMV-luc as described [45]. The acquired reAc-luc was then purified via a round of plaque purification [44]. Sf9 cells were infected with the purified virus at 0.1 MOI (multiplicity of infection of 0.1 p.f.u. per cell). Four days after infection, the larval hemolymph and cell-culture fluid were collected. The viruses were then purified and concentrated by centrifugation at 100,000 g for 80 min at 4°C through a 30% (w/v) sucrose cushion [27].

### Quantitative polymerase chain reaction (Q-PCR) analysis of the viral genome

Using the recombinant viruses as templates and primers (viral genome primers, VG-QF: GACA CCGAAACTCCGTATTGCC, VG-QR: ATCCGTTGA TTCCGTTGACACC; luc-QF: GGTGGACATCACTTA CGC, luc-QR: AATGCCCATACTGTTGAG; EGFP-QF: CACAAGTTCAGCGTGTCCG, EGFP-QR: CTCGATGCGGTTCACCAG), Q-PCR was performed using the Toyobo SYBR Green. Standard curves were constructed based on serial dilutions of PCR products that ranged from 10^12^ to 10^6^ copies per microliter. The purified recombinant baculovirus solutions were quantified based on the Q-PCR results as the amount of virus genome per milliliter (vg/mL) [46]. The quantified results of the luciferase and EGFP reporter genes were compared with the viral genome results.

### Complement inactivation assay of baculoviruses

VERO cells were cultured in 6-well plates at a constant cell density of 1×10^6^ cells per well. Human serum was pretreated as described [27]. Purified reBm-luc or reAc-luc vectors with an MOI of 100 in the infection assay were incubated with different concentrations of human serum at 37°C for 30 min, and then added to VERO cells for infection. Luciferase activity was measured 42 h later. The survival of the baculovirus vector was measured as the percentage of luciferase activity, which was compared with human serum heat-treated at 56°C as a control [27].

### Delivery of reporter genes *in vitro*

VERO, PK-15, HEK293T, and Hela cells were cultured in 6-well plates at a constant cell density of 1×10^6^ cells per well. Purified reBm-EGFP with an MOI of 100 was added into cultured cells for transduction. The treated cells were observed under an inverted fluorescence microscope (Nikon, Japan) 48 h later [12].

### Delivery of reporter genes *in vivo*

The recombinant *Bm*NPVs, reBm-EGFP and reBm-Luc vectors were used for gene delivery. Wild-type *Bm*NPV containing no reporter gene was used as a control. Intravenous injection group (IV): C57BL/6 mice were infected with control *Bm*NPV or recombinant *Bm*NPVs containing recombinant reporter genes via tail vein injection at 1×10^12^ viral genome per kilogram (vg/kg); chicks were infected via wing vein injection at 1×10^12^ vg/kg. Intramuscular injection group (IM): C57BL/6 mice and chicks were infected with control or delivery *Bm*NPVs via injection in the muscle of the quadriceps femoris or hind limb at 1×10^12^ vg/kg. Intragastric administration group (IG): C57BL/6 mice and chicks were starved for 12 h before gavage infusion with control or delivery *Bm*NPVs at 1×10^13^ vg/kg. Three mice and three chicks were sacrificed by bloodletting 5, 11, 17, 21 and 30 d after recombinant baculovirus administration. Organs were excised for reporter gene assays. The EGFP-transfected organs were sectioned on a freezing microtome (CM3050S, Leica, Germany) or imaged by fluorescence microscope (Nikon, Japan). Homogenates (50 μg of protein) prepared from the reBm-luc treated organs were assayed using a Luciferase Assay kit (Promega). The amount of protein in the homogenates was measured using the Bradford method [47].

## SUPPLEMENTARY MATERIALS FIGURE


