# Supplementary material for: Gene delivery and gene expression in vertebrate using baculovirus Bombyx mori nucleopolyhedrovirus vector
Source: Oncotarget. 2017 Nov 20;8(62):106017–25. doi: 10.18632/oncotarget.22522 (PMC5739698; doi:10.18632/oncotarget.22522)
Supplement: Supplementary file 1 [file oncotarget-08-106017-s001.pdf]

Gene delivery and gene expression in vertebrate using baculovirus *Bombyx mori* nucleopolyhedrovirus vector

SUPPLEMENTARY MATERIALS

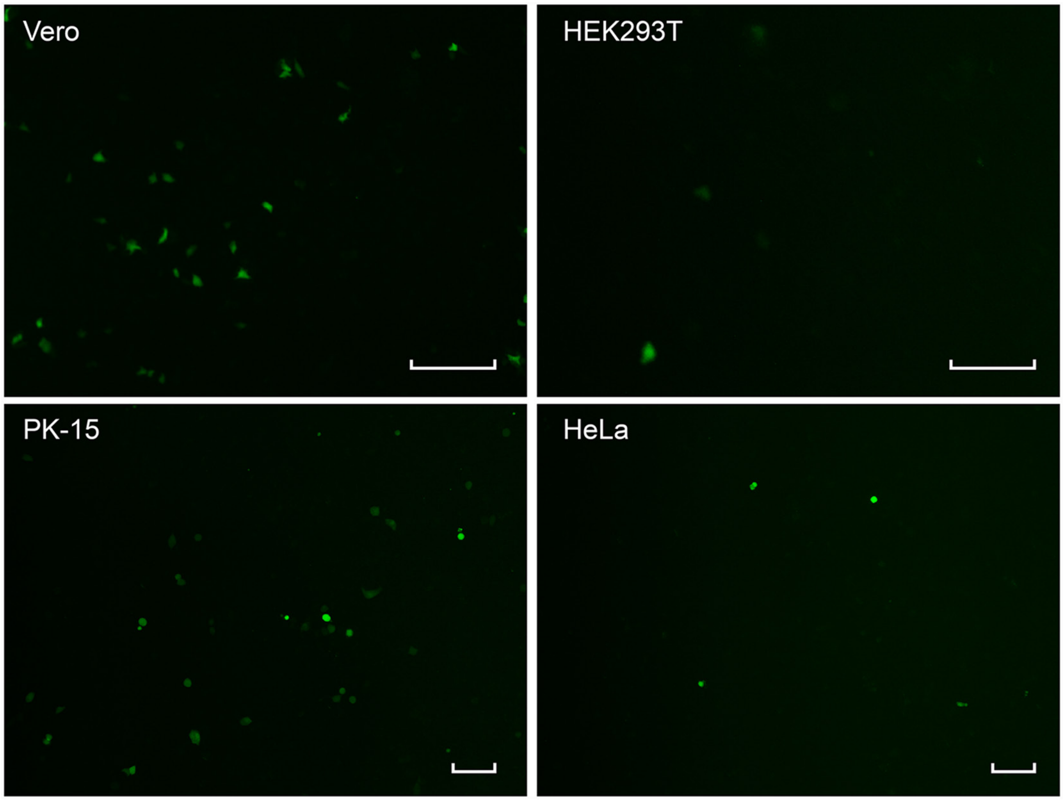

Supplmentary Figure 1: Expression of EGFP in various mammalian cells transduced with reBm-EGFP. Scale bar, 100 µm.
